# Discovery of Tyrosinase Inhibitors from *Lysinibacillus* sp. JNUCC 52 via Genome Mining, Secondary Metabolites Profiling, and In Silico Analysis

**DOI:** 10.3390/cimb48030280

**Published:** 2026-03-05

**Authors:** Xuhui Liang, Yang Xu, Chang-Gu Hyun

**Affiliations:** Department of Chemistry and Cosmetics, Jeju National University, Jeju 63243, Republic of Korea; lxh03036@gmail.com (X.L.); iamxuyang1990@gmail.com (Y.X.)

**Keywords:** *Lysinibacillus* sp. JNUCC 52, genome annotation, secondary metabolites, tyrosinase inhibitor, molecular docking, molecular dynamics simulations

## Abstract

Tyrosinase is a key enzyme in melanin biosynthesis, and natural inhibitors have potential therapeutic and cosmetic applications. *Lysinibacillus* sp. JNUCC 52, a member of the Bacillaceae family, shows potential for producing bioactive secondary metabolites. However, the tyrosinase inhibitory potential of metabolites from this strain has not been previously reported. This study investigates its genomic features, secondary metabolites, and tyrosinase inhibitory activity to identify promising enzyme inhibitors. Integrated COG, GO, and KEGG annotation revealed a metabolically robust network supporting secondary metabolite biosynthesis. Chemical investigation of the ethyl acetate extract yielded five known compounds, among which cyclo(*L*-Pro-*L*-Leu) displayed the strongest tyrosinase inhibition (IC_50_ = 79.5 ± 2.3 μM), whereas uracil showed weaker activity. In silico ADMET and drug-likeness analyses suggested favorable pharmacokinetic properties and compliance with major drug-likeness rules for cyclo(*L*-Pro-*L*-Leu). Molecular docking and molecular dynamics simulations demonstrated stable binding to mushroom tyrosinase (mTYR) and human TYRP1, supported by MM/GBSA and residue decomposition analyses identifying key stabilizing residues. Together, these results provide mechanistic insight into tyrosinase inhibition and highlight cyclo(*L*-Pro-*L*-Leu) as a minimal lead-like scaffold, while establishing strain JNUCC 52 as a promising microbial source of bioactive metabolites.

## 1. Introduction

*Lysinibacillus* is a spore-forming bacterial genus reclassified from the genus *Bacillus* based on distinctive cell wall peptidoglycan composition and phylogenetic analyses of 16S rRNA gene sequences, including the reassignment of *Bacillus sphaericus* and *Bacillus fusiformis* [1]. Members of this genus are characterized by the A4a (Lys–Asp) type peptidoglycan, motile rod-shaped cells, and the ability to form spherical or ellipsoidal endospores, conferring strong environmental adaptability and stable cultivability [2,3]. As a member of the family Bacillaceae, *Lysinibacillus* represents a potentially valuable microbial resource. Previous studies have demonstrated that *Lysinibacillus* species exhibit diverse biological functions, including biological control of agricultural pests, heavy metal remediation, and plant growth promotion, suggesting that their ecological roles may be closely associated with the production of secondary metabolites [4,5]. Indeed, this genus produces a variety of bioactive compounds, including indole-3-acetic acid (IAA), siderophores, extracellular enzymes, antimicrobial metabolites, bacteriocins, and nematicidal volatiles, which collectively contribute to its ecological functions [6,7].

Members of the family Bacillaceae are prolific producers of bioactive secondary metabolites, including cyclic lipopeptides, azole-containing peptides, and nonribosomal peptide antibiotics, many of which exhibit antibacterial, antifungal, enzyme-inhibitory, or other bioactive properties. Bacitracin, a cyclic peptide antibiotic, inhibits bacterial peptidoglycan biosynthesis by forming a stable complex with undecaprenyl pyrophosphate (C_55_-PP), thereby preventing its enzymatic dephosphorylation and recycling of the lipid carrier essential for cell wall assembly [8,9]. Beyond antibacterial targets, certain *Bacillaceae*-derived metabolites also exhibit biologically relevant activities in mammalian systems. Surfactin, a bacterial cyclic lipopeptide, induces apoptosis and cell-cycle arrest in human carcinoma cells via ROS-mediated JNK/mitochondrial/caspase pathways [10,11]. It also modulates NF-κB and MAPK/Akt signaling in macrophages, exhibiting anti-inflammatory and immunoregulatory effects [12,13], and reduces pro-inflammatory mediator production in microglia via HO-1/Nrf-2 signaling, suggesting neuroprotective potential [14], supporting its multifunctional bioactivity. Ribosomally synthesized azole-containing peptides such as plantazolicin, isolated from *Bacillus amyloliquefaciens* FZB42, display highly selective antibacterial activity, including inhibition of *Bacillus anthracis*, illustrating the presence of narrow-spectrum antibiotic natural products in this family [15,16]. Bacilysin, a non-ribosomal dipeptide antibiotic produced by certain *Bacillus* strains, exhibits broad antimicrobial activity against a range of bacteria and fungi and has been characterized for its biosynthesis and mode of action in microbial systems [17].

Although Bacillaceae are widely acknowledged for producing enzyme-modulating secondary metabolites, comparatively few studies have focused on the fermentation-derived metabolites of the genus *Lysinibacillus*. Tyrosinase (EC 1.14.18.1), a copper-containing oxidase, catalyzes the initial steps of melanin biosynthesis and drives pigmentation in mammalian skin as well as enzymatic browning in fruits and vegetables, making it a key target in cosmetic and food industry research [18,19]. Consequently, identifying safe and effective tyrosinase inhibitors continues to be a central focus of natural product research. Notably, several well-established tyrosinase inhibitors have been isolated from microbial sources, including kojic acid produced by *Aspergillus* species [20], azelaic acid derived from *Malassezia* spp. [21], and tropolone reported from *Streptomyces* species [22]. Cyclo(*L*-Pro-*L*-Tyr) (maculosin), a naturally occurring 2,5-diketopiperazine (DKP) derivative, has also been reported to inhibit tyrosinase activity and melanin production in B16F10 melanoma cells [23], highlighting that microorganisms are a validated yet underexplored source of tyrosinase inhibitory compounds.

In this study, *Lysinibacillus* sp. JNUCC 52, isolated from Baengnokdam, the summit crater of Hallasan Mountain, was subjected to fermentation, and its secondary metabolites were systematically isolated, structurally characterized, and evaluated for tyrosinase inhibition using enzymatic assays in combination with molecular docking and molecular dynamics simulations. This work contributes to the identification and characterization of microbially derived tyrosinase inhibitors and provides a basis for exploring their potential applications in pharmaceutical, cosmetic, and food research.

## 2. Materials and Methods

### 2.1. Genome Sequencing and Analysis of Strain JNUCC 52

The strain JNUCC 52 analyzed in this study was isolated from soil collected in September 2019 at Baengnokdam, the summit crater of Hallasan Mountain, Jeju, Korea. To recover viable microorganisms, 0.5 g of soil was suspended in 0.45 mL of 0.1% Tris solution and incubated at 30 °C with shaking at 180 rpm for 1 h. A 100 μL aliquot of the resulting suspension was serially diluted from 10^−5^ to 10^−9^ and plated on MRS agar for colony development.

Bacterial colonies were routinely maintained under aerobic conditions on LB agar plates or in LB broth at 30 °C for approximately 24 h. For long-term storage, cultures in the exponential growth phase were mixed with glycerol to a final concentration of 20% and stored at −80 °C. Single-colony genomic DNA was extracted using a QIAGEN Genomic-tip kit (Qiagen Inc., Shenzhen, China). Whole-genome sequencing was performed using a hybrid PacBio RS II and Illumina platform at Macrogen (Seoul, Republic of Korea).

### 2.2. Phylogenetic Tree Construction

To determine the evolutionary placement of strain JNUCC 52, genome assemblies of taxonomically related species were retrieved from the NCBI database. Orthologous gene clusters, including single-copy orthologs, were identified using OrthoFinder v2.5.5. Single-copy orthologous genes were individually aligned with MUSCLE v5.1, and the resulting alignments were concatenated for phylogenetic analysis. Phylogenetic trees were constructed using the maximum-likelihood method implemented in IQ-TREE v2.2.2.7. Branch support was evaluated based on 1000 bootstrap replicates.

### 2.3. Functional Genome Annotation

Functional annotation of predicted coding sequences was performed using BLAST+ (v2.12.0), BLAST2GO (v5.2), and DIAMOND (v2.0.15) searches. Annotated genes were assigned to the COG, GO, and KEGG databases to infer functional categories and metabolic pathways. DIAMOND alignments were conducted with an E-value threshold of 1 × 10^−5^ to ensure high-confidence annotations.

### 2.4. Fermentation, Extraction, and Compound Identification of Lysinibacillus sp. JNUCC 52

The strain JNUCC 52 was cultured in 250 mL flasks containing 125 mL LB medium at 30 °C with agitation for 48 h. Four 5 L LB flasks containing a total of 4 L medium were inoculated with the seed culture at 0.5% (*v*/*v*) and incubated aerobically at 30 °C for 4 days. After filtration through paper to remove biomass, 4 L of cell-free supernatant was extracted three times with 2 L portions of ethyl acetate. The combined organic phase was concentrated under reduced pressure to yield 580 mg crude extract (HPLC profile shown in Figure S1, Table S1), which was fractionated on silica gel using a stepwise CHCl_3_–MeOH gradient (300 mL per fraction), producing ten fractions (Fr. V1–V10). Compound **1** (11.3 mg, RT 8.4 min, 98.83% purity; Figure S2a) was purified from Fr. V4 by silica gel column chromatography using CHCl_3_–MeOH (50:1, *v*/*v*). Under the same conditions, Fr. V5 yielded compound **2** (20.0 mg) and compound **3** (7.8 mg, RT 5.5 min, 89.23% purity; Figure S2b), while Fr. V7 afforded compound **4** (9.0 mg, RT 26.499 min, 98.26% purity; Figure S2c) and compound **5** (7.0 mg, RT 2.56 min, 87.92% purity; Figure S2d).

Compound **1**, cyclo(*L*-Pro-*L*-Tyr). ^1^H NMR (500 MHz, METHANOL-*D_4_*) δ 7.04–6.98 (m, 2H), 6.71–6.64 (m, 2H), 4.33 (td, *J* = 4.8, 1.9 Hz, 1H), 4.02 (ddd, *J* = 10.9, 6.3, 2.0 Hz, 1H), 3.52 (dt, *J* = 11.9, 8.3 Hz, 1H), 3.32 (dt, *J* = 12.5, 6.6 Hz, 2H), 3.06 (dd, *J* = 14.2, 5.1 Hz, 1H), 3.00 (dd, *J* = 14.2, 4.6 Hz, 1H), 2.11–2.02 (m, 1H), 1.83–1.73 (m, 2H). ^13^C NMR (126 MHz, METHANOL-*D*_4_) δ 170.78, 166.96, 157.68, 132.13, 127.62, 116.18, 60.05, 57.90, 45.91, 37.66, 29.39, 22.71 (Figures S3 and S4) [24].

Compound **2**, ethyl laurate. ^1^H NMR (500 MHz, CHLOROFORM-*D*) δ 4.11 (qd, *J* = 7.1, 0.6 Hz, 1H), 2.03 (d, *J* = 0.6 Hz, 1H), 1.82–1.64 (m, 1H), 1.35–1.18 (m, 54H), 0.92–0.77 (m, 21H). ^13^C NMR (126 MHz, CHLOROFORM-*D*) δ 171.30, 60.51, 37.19, 32.84, 32.02, 30.13, 29.80, 29.46, 27.18, 22.79, 14.28, 14.22 (Figures S5 and S6) [25].

Compound **3**, cyclo(*L*-Pro-*L*-Leu). ^1^H NMR (500 MHz, CHLOROFORM-*D*) δ 4.15–4.09 (m, 1H), 4.06–3.92 (m, 1H), 3.65–3.49 (m, 2H), 2.34 (dtd, *J* = 13.0, 6.8, 2.9 Hz, 1H), 2.11 (dtd, *J* = 12.6, 10.0, 7.2 Hz, 1H), 2.02 (ddt, *J* = 16.5, 7.3, 3.8 Hz, 1H), 1.94–1.70 (m, 3H), 1.69–1.53 (m, 1H), 0.98 (m, 3H), 0.96–0.90 (m, 3H). ^13^C NMR (126 MHz, CHLOROFORM-*D*) δ 170.54, 166.47, 59.38, 51.28, 45.56, 38.59, 28.25, 23.43, 23.39, 22.87, 21.08 (Figures S7 and S8) [26].

Compound **4**, indole. ^1^H NMR (500 MHz, CHLOROFORM-*D*) δ 7.72–7.62 (m, 1H), 7.40 (dq, *J* = 8.1, 1.0 Hz, 1H), 7.24–7.15 (m, 2H), 7.12 (ddd, *J* = 8.0, 7.0, 1.0 Hz, 1H), 6.56 (ddd, *J* = 3.1, 2.1, 1.0 Hz, 1H). ^13^C NMR (126 MHz, CHLOROFORM-*D*) δ 135.86, 127.93, 124.21, 122.08, 120.82, 119.90, 111.10, 102.72, 11.06 (Figures S9 and S10) [27].

Compound **5**, uracil. ^1^H NMR (500 MHz, DMSO-*D*_6_) δ 7.35 (d, *J* = 7.6 Hz, 1H), 5.40 (d, *J* = 7.6 Hz, 1H). ^13^C NMR (126 MHz, DMSO-*D*_6_) δ 164.86, 152.03, 142.76, 100.72 (Figures S11 and S12) [28].

### 2.5. Mushroom Tyrosinase Inhibition Assay

Tyrosinase inhibition was measured spectrophotometrically using L-tyrosine as substrate. Each well of a 96-well plate received 20 μL of test compound, followed by 130 μL of 2 mM L-tyrosine in 0.1 M potassium phosphate buffer (pH 6.8), 5 μL tyrosinase (2500 U), and 45 μL buffer to initiate the reaction. Following incubation at 37 °C for 10 min, the formation of dopachrome was monitored by measuring absorbance at 490 nm using a microplate reader. Arbutin was employed as standard inhibitors for comparative analysis. IC_50_ values were determined based on three independent experimental replicates.

### 2.6. Molecular Properties and Drug Likeness Analysis

ADMET and Drug-likeness properties were evaluated through in silico prediction. The analysis was conducted using multiple established platforms, including ADMETlab 3.0 (https://admetlab3.scbdd.com/, accessed on 5 December 2025), SwissADME (http://www.swissadme.ch/, accessed on 5 December 2025), and pkCSM (https://biosig.lab.uq.edu.au/pkcsm/, accessed on 5 December 2025).

### 2.7. Molecular Docking Simulations Analysis

The 3D structures of mushroom tyrosinase (PDB ID: 2Y9X) and human TYRP1 (PDB ID: 5M8M) were obtained from the Protein Data Bank and processed in PyMOL (v2.5.5) to verify structural integrity. Ligand structures were obtained from PubChem (https://pubchem.ncbi.nlm.nih.gov/, accessed on 5 December 2025) and energy-minimized using OpenBabel (v3.1.1) with the MMFF94 force field.

Proteins were prepared in AutoDock Tools (v1.5.7) by adding polar hydrogens and assigning atom types, while ligands were assigned hydrogens and rotatable bonds. Docking grids were centered on the binding sites defined by the co-crystallized inhibitors. The docking protocol was previously validated by redocking the native ligands into both protein structures, yielding RMSD values below 2.0 Å, thereby confirming the reliability of the docking setup [29]. Docking was performed with AutoDock Vina in semi-flexible mode (exhaustiveness = 25), employing the Lamarckian Genetic Algorithm [30], and ligand–protein interactions were analyzed using Discovery Studio 2019.

### 2.8. Molecular Dynamics Simulations Analysis

Molecular dynamics simulations were carried out using GROMACS 2021. Proteins were parameterized with AMBER14SB and ligands with GAFF2. Each protein–ligand complex was solvated in a TIP3P water box with 1.2 nm padding, neutralized with Na^+^/Cl^−^ ions, and adjusted to 0.15 M ionic strength. Systems were energy-minimized using the steepest-descent algorithm for 50,000 steps (convergence threshold 100 kJ·mol^−1^·nm^−1^). Equilibration involved 1 ns NVT (V-rescale, T = 310 K, τ = 0.1 ps). This was followed by a 1 ns NPT equilibration using a Parrinello–Rahman barostat (1 atm, τ = 1 ps) [31], with 1000 kJ·mol^−1^·nm^−2^ restraints on heavy atoms. Production runs of 100 ns were performed in the NPT ensemble without restraints, using periodic boundary conditions, Particle Mesh Ewald for long-range electrostatics [32], 1.2 nm cutoffs for short-range interactions, LINCS constraints on bonds involving hydrogen [33], and a 2 fs timestep. Each system was simulated in triplicate to ensure reproducibility.

The FEL was generated using g_sham in GROMACS, with RMSD and radius of Rg as reaction coordinates. MD trajectories were corrected for periodic boundary conditions and analyzed using Visual Molecular Dynamics (VMD). PCA and DCCM analyses were performed with the Bio3D package in R (v4.3.1). Binding free energies were calculated using the MM/GBSA method implemented in gmx_MM/PBSA [34] based on the final 30 ns of equilibrated trajectories. Residue energy decomposition was conducted for residues within 4 Å of the ligand to identify key contributors to binding.

## 3. Results and Discussion

### 3.1. General Features of Strain JNUCC 52 Genome

**Figure 1:**
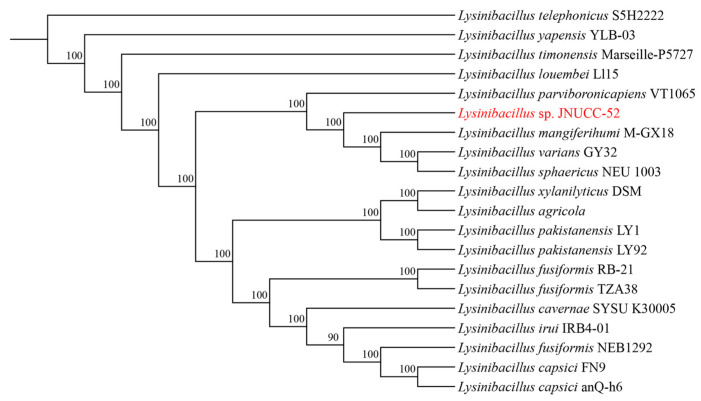
Phylogenomic tree of *Lysinibacillus* sp. JNUCC 52.

Whole-genome sequencing of *Lysinibacillus* sp. JNUCC 52 generated 4,681,075 bp of high-quality sequences with a GC content of 37%. The complete genome comprises 4456 coding sequences (CDSs), representing 96.04% of the total genes, along with 112 tRNA genes and 37 rRNA genes (13 copies of 5S, 12 copies of 16S, and 12 copies of 23S). The assembled genome sequence has been deposited in the NCBI GenBank database under the accession number NZ_CP065546. Phylogenomic analysis based on single-copy orthologous genes revealed that strain JNUCC 52 is closely related to *Lysinibacillus* mangiferhumi M-GX18, indicating a close evolutionary relationship within the genus *Lysinibacillus* (Figure 1).

### 3.2. General Genome Annotation of Strain JNUCC 52

#### 3.2.1. COG Annotation

**Figure 2:**
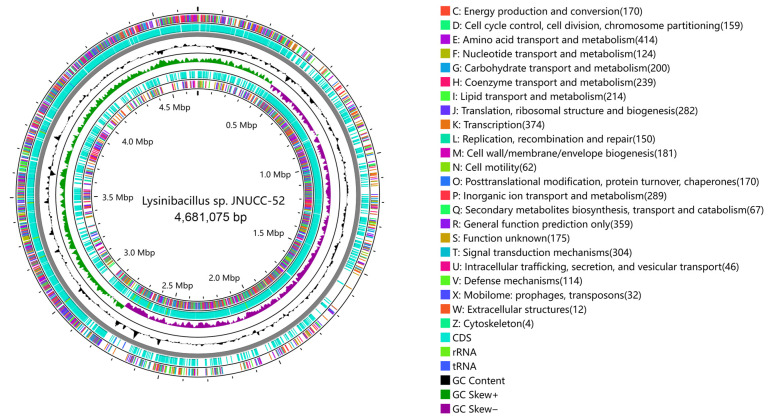
COG classification of the strain JNUCC 52 genome.

A total of 4456 protein-coding sequences were identified in the *Lysinibacillus* sp. JNUCC 52 genome, of which 3635 were assigned to clusters of orthologous groups (COGs) (Figure 2). The predominant COG functional categories (each accounting for ≥5% of all classified ORFs) included amino acid transport and metabolism (E; 414 ORFs, 11.39%), transcription (K; 374 ORFs, 10.29%), general function prediction only (R; 359 ORFs, 9.88%), signal transduction mechanisms (T; 304 ORFs, 8.37%), inorganic ion transport and metabolism (P; 289 ORFs, 7.95%), translation, ribosomal structure, and biogenesis (J; 282 ORFs, 7.76%), coenzyme transport and metabolism (H; 239 ORFs, 6.57%), lipid transport and metabolism (I; 214 ORFs, 5.89%), and carbohydrate transport and metabolism (G; 200 ORFs, 5.50%).

#### 3.2.2. GO Annotation

**Figure 3:**
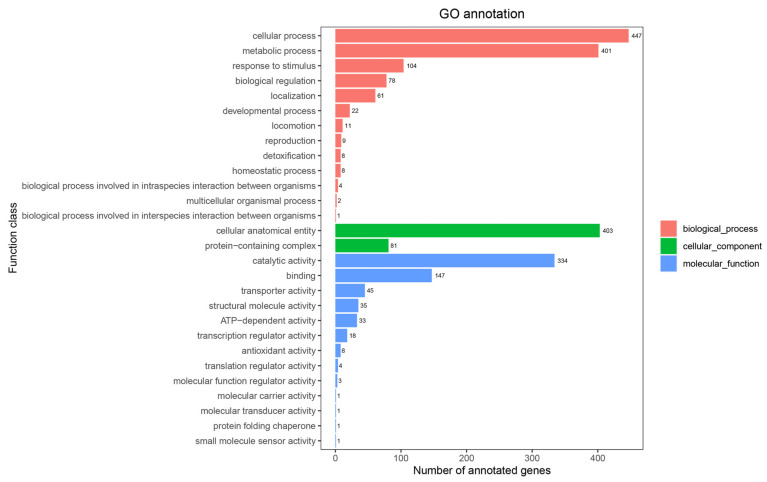
GO categories of the strain JNUCC 52 genome.

A total of 2271 genes were annotated in the Gene Ontology (GO) database, representing 51.0% of the total coding sequences in *Lysinibacillus* sp. JNUCC 52 (Figure 3). Among the three major GO categories, biological process was the most represented (gene number: 1156), followed by molecular function (gene number: 631) and cellular component (gene number: 484). Within the biological process category, genes associated with cellular process (gene number: 447) and metabolic process (gene number: 401) were predominant. In the molecular function category, catalytic activity (gene number: 334) and binding (gene number: 147) were the most abundant subcategories, while cellular anatomical entity (gene number: 403) dominated the cellular component category.

#### 3.2.3. KEGG Annotation

**Figure 4:**
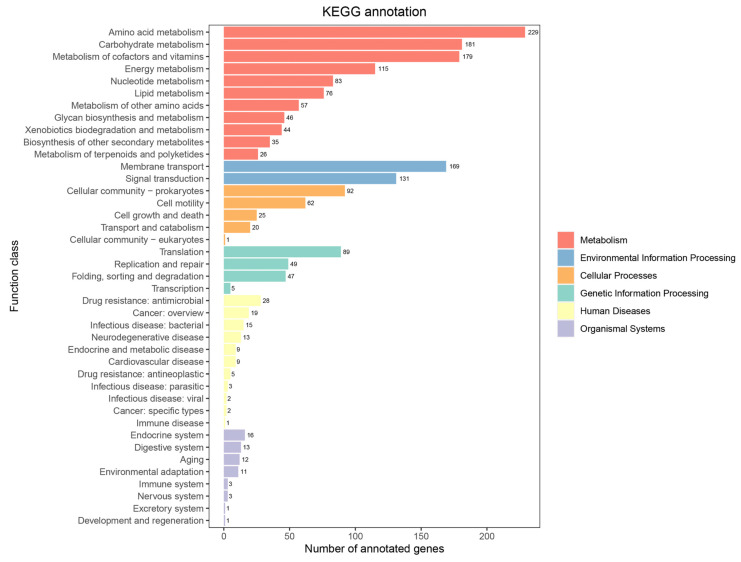
KEGG pathways of the strain JNUCC 52 genome.

According to the Kyoto Encyclopedia of Genes and Genomes (KEGG) database, the genome of the strain JNUCC 52 harbored a broad spectrum of functional genes associated with diverse metabolic and cellular pathways (Figure 4). A total of 1028 genes were assigned to the metabolism category, including amino acid metabolism (gene number: 229), carbohydrate metabolism (gene number: 181), metabolism of cofactors and vitamins (gene number: 179), energy metabolism (gene number: 115), nucleotide metabolism (gene number: 83), and lipid metabolism (gene number: 76). In addition, 300 genes were annotated under the environmental information processing category, comprising membrane transport (gene number: 169) and signal transduction (gene number: 131). Furthermore, 200 genes were assigned to the cellular processes category, including cellular community–prokaryotes (gene number: 92) and cell motility (gene number: 62). A total of 190 genes were involved in genetic information processing, primarily associated with translation (gene number: 89) and replication and repair (gene number: 49).

### 3.3. Secondary Metabolites Isolated from the Strain JNUCC 52

**Figure 5:**
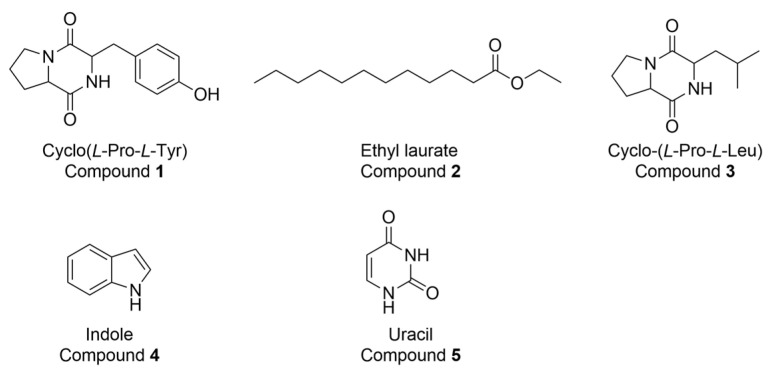
Chemical structure of the isolated compounds.

Purification of the ethyl acetate-soluble fraction (580 mg) of the fermentation crude extract from the culture broth of strain JNUCC52 afforded five known compounds: cyclo(*L*-Pro-*L*-Tyr), ethyl laurate, cyclo(*L*-Pro-*L*-Leu), indole, and uracil (Figure 5). These compounds, although reported in other microbial species, expand the known secondary metabolite repertoire of *Lysinibacillus* spp. within the family Bacillaceae. Cyclo(*L*-Pro-*L*-Tyr) and cyclo(*L*-Pro-*L*-Leu) are 2,5-DKP derivatives, the simplest cyclic dipeptides, which are widely distributed in microorganisms, plants, and animals [35,36,37,38,39]. DKPs are biosynthesized via nonribosomal peptide synthetases (NRPSs) or cyclodipeptide synthases from two α-amino acids, with additional contributions from proteolytic or enzymatic pathways [40,41,42]. In bacteria such as *Pseudomonas aeruginosa*, NRPS proteins have been shown to mediate the formation of cyclic dipeptides, including cyclo(*L*-Pro-*L*-Leu) [43]. Although no cyclic dipeptide biosynthetic gene cluster was identified by antiSMASH, genome annotation of strain JNUCC52 indicated genes associated with amino acid and peptide metabolism. As simple DKPs may also arise from NRPS-independent or alternative enzymatic routes in bacteria, cyclic dipeptide production in this strain remains plausible despite the absence of a canonical gene cluster.

### 3.4. mTYR Inhibitory Activities Analysis

**Figure 6:**
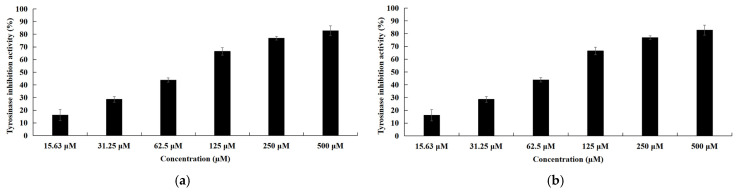
Tyrosinase inhibitory activities of the isolated compounds. (**a**) cyclo(*L*-Pro-*L*-Leu) and (**b**) uracil.

Among the isolated compounds, cyclo(*L*-Pro-*L*-Leu) exhibited tyrosinase inhibitory activity (IC_50_ = 79.5 ± 2.3 μM; Figure 6a), while arbutin showed an IC_50_ value of 157.7 ± 2.1 μM under identical assay conditions; however, arbutin is a well-established tyrosinase inhibitor in cosmetic applications, whereas the activity of cyclo(*L*-Pro-*L*-Leu) remains to be further explored. Natural tyrosinase inhibitors typically display IC_50_ values spanning the low to several hundred micromolar range [44,45]. Accordingly, cyclo(*L*-Pro-*L*-Leu) can be classified as a moderately active inhibitor. In contrast, uracil showed relatively weak inhibition, with an IC_50_ value of 596 ± 3.2 μM (Figure 6b). Cyclic dipeptides are known to exhibit diverse bioactivities, including antibacterial, antifungal, antitumor, quorum-sensing modulation, and antifouling effects, and their stable scaffold renders them promising candidates for drug development [46,47,48]. The tyrosinase inhibitory activity of cyclo(*L*-Pro-*L*-Tyr) has been previously reported, whereas cyclo(*L*-Pro-*L*-Leu) is reported for the first time as a mTYR inhibitor. The remaining two compounds did not display detectable tyrosinase inhibitory activity under the assay conditions.

### 3.5. In Silico Analysis

#### 3.5.1. ADMET and Drug-Likeness Evaluation

As summarized in Table S2, the ADMET (Absorption, Distribution, Metabolism, Excretion, and Toxicity) profiles of the four compounds (cyclo(*L*-Pro-*L*-Tyr), cyclo(*L*-Pro-*L*-Leu), uracil, and arbutin) were evaluated. Among them, cyclo(*L*-Pro-*L*-Leu) was predicted to exhibit a comparatively balanced pharmacokinetic profile, with a moderate-to-high predicted human intestinal absorption (84.24%) and no predicted liability as a P-gp substrate or inhibitor, suggesting that its bioavailability may be less affected by efflux transport mechanisms. The predicted plasma protein binding of cyclo(*L*-Pro-*L*-Leu) was relatively low (24.1%), and no tendency for blood–brain barrier penetration was predicted. Uracil was predicted to show the highest intestinal absorption and minimal plasma protein binding; however, its relatively high predicted clearance may limit systemic exposure. In contrast, Arbutin was predicted to have lower intestinal absorption (38.03%) and higher plasma protein binding, indicating potentially limited oral absorption and distribution. Metabolic liability assessment suggested that none of the compounds were predicted to inhibit major CYP450 isoforms, implying a low risk of CYP-mediated drug–drug interactions. Computational toxicity profiling revealed no predicted hepatotoxicity, mutagenicity, skin sensitization, or hERG inhibition for cyclo(*L*-Pro-*L*-Leu), uracil, and arbutin, while cyclo(*L*-Pro-*L*-Tyr) was associated with a predicted hepatotoxic risk.

Based on the drug-likeness assessment (Table S3), cyclo(*L*-Pro-*L*-Tyr) and cyclo(*L*-Pro-*L*-Leu) satisfy all evaluated drug-likeness rules, whereas uracil and arbutin do not meet the Ghose filter but remain compliant with Lipinski’s Rule of Five, the Veber rule, and the Egan rule. Notably, the Ghose criteria were developed for oral pharmaceuticals and are not necessarily restrictive for cosmetic or topical agents, as illustrated by the widespread cosmetic use of arbutin. Overall, the in silico ADMET and drug-likeness predictions suggest that the four compounds display distinct pharmacokinetic and physicochemical characteristics, with cyclo(*L*-Pro-*L*-Leu) showing a relatively favorable predicted profile, while Uracil and Arbutin exhibit specific limitations in drug-likeness filters.

#### 3.5.2. Molecular Docking Simulations

**Figure 7:**
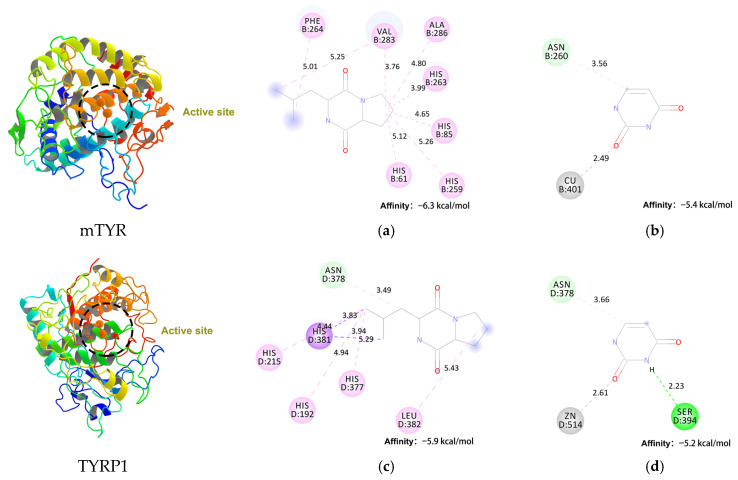
Molecular docking analysis of bioactive compounds with tyrosinase. Visualized binding interactions of (**a**,**c**) Cyclo(*L*-Pro-*L*-Leu), (**b**,**d**) Uracil with mTYR (**a**,**b**) and TYRP1 (**c**,**d**), respectively. Hydrogen bonds are shown in green, C–H bonds in light green, alkyl or π–alkyl interactions in pink, π–σ interactions in purple, and metal–acceptor interactions in gray.

To elucidate the inhibitory mechanism of cyclo(*L*-Pro-*L*-Leu) and uracil against tyrosinase, molecular docking studies were performed for both compounds with mushroom tyrosinase (mTYR, PDB ID: 2Y9X) and human tyrosinase related protein 1 (TYRP1, PDB ID: 5M8M) (Figure 7). Cyclo(*L*-Pro-*L*-Leu) exhibited a binding energy of −6.3 kcal/mol toward mTYR, substantially stronger than that of uracil at −5.4 kcal/mol. A similar trend was observed for TYRP1, with binding energies of −5.9 kcal/mol and −5.2 kcal/mol, respectively. These results correlate well with the experimentally observed differences in mTYR inhibition.

Cyclo(*L*-Pro-*L*-Leu) interacts with both enzymes primarily through hydrophobic interactions. In mTYR, the cyclic dipeptide establishes extensive interactions with His61, His85, His259, His263, Phe264, Val283, and Ala286, while in TYRP1, interactions involve His192, His215, His377, Asn378, His381, and Leu382. This hydrophobic complementarity facilitates optimal occupancy of the catalytic pocket, stabilizing the ligand and accounting for its favorable binding energy. Previous reports suggested that maculosin forms hydrogen-bonding and π-related interactions [23], whereas cyclo(*L*-Pro-*L*-Leu) is primarily stabilized through hydrophobic contacts. Molecular docking analyses indicated that both maculosin and cyclo(*L*-Pro-*L*-Leu) occupy partially overlapping regions within the catalytic pockets of mTYR and TYRP1. In the predicted binding poses, both ligands interact with key residues such as His263 and Val283 in mTYR, as well as His381 in TYRP1.

In contrast, uracil exhibits a metal-dependent binding mode, coordinating its carbonyl oxygen to the catalytic Cu^2+^ in mTYR and Zn^2+^ in TYRP1 at distances of 2.49 Å and 2.61 Å, respectively, consistent with monodentate carbonyl–metal coordination. These interactions are generally considered moderate-strength anchoring interactions, dominated by σ donation with limited π back-donation, and do not correspond to strong or multidentate metal chelation [49,50,51]. The paucity of additional functional groups further limits stabilizing interactions, which are mainly observed with Asn260 in mTYR and Ser394 in TYRP1. Consequently, uracil exhibits weaker overall binding compared with cyclo(*L*-Pro-*L*-Leu).

#### 3.5.3. Molecular Dynamics Simulations

**Figure 8:**
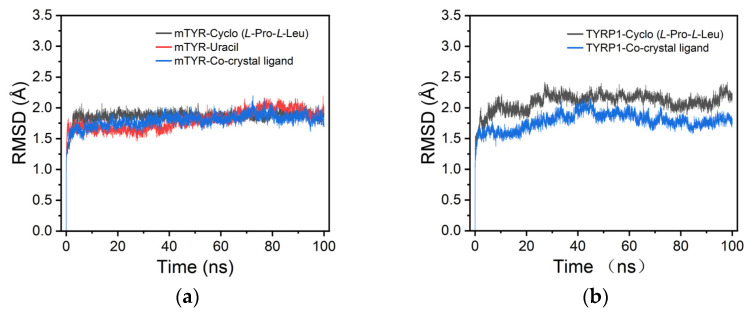
RMSD analysis of protein–ligand complexes. (**a**) mTYR–ligand complexes; (**b**) TYRP1–ligand complexes.

For mTYR, the RMSD analysis showed that both cyclo(*L*-Pro-*L*-Leu) and uracil maintained relatively stable conformations within the binding site, with RMSD values consistently remaining in the range of 1.5–2.0 Å, indicating stable ligand binding to mTYR (Figure 8a). For TYRP1, the RMSD profile of cyclo(*L*-Pro-*L*-Leu) closely resembled that of the co-crystallized ligand, remaining stable throughout the simulation within 1.5–2.5 Å, suggesting reliable binding stability (Figure 8b).

Analysis of representative snapshots from the MD simulations showed that the ligands exhibited stable binding to the mTYR protein (Figure S13). By contrast, uracil rapidly dissociated from the binding pocket at the early stage of the simulation, indicating a lack of stable complex formation with TYRP1. This difference in binding behavior may result from the structurally simple nature of uracil, which lacks sufficient interaction sites to maintain stable binding.

**Figure 9:**
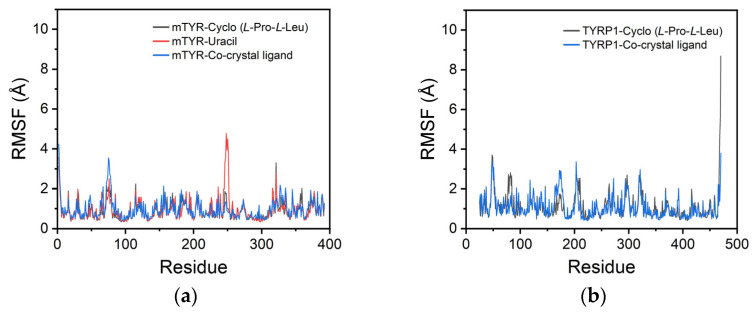
RMSF analysis of protein. (**a**) mTYR; (**b**) TYRP1.

For mTYR, the RMSF profiles of the cyclo(*L*-Pro-*L*-Leu) and uracil complexes closely overlapped with the co-crystallized inhibitor, with overall fluctuations below 10 Å, indicating that ligand binding did not significantly alter the protein conformation (Figure 9a). Notably, uracil exhibited a localized fluctuation of approximately 5 Å near residue 250, likely corresponding to a flexible region of the protein. For TYRP1, the RMSF profile of cyclo(*L*-Pro-*L*-Leu) closely matched the co-crystallized inhibitor, indicating stable binding throughout the simulation (Figure 9b).

**Figure 10:**
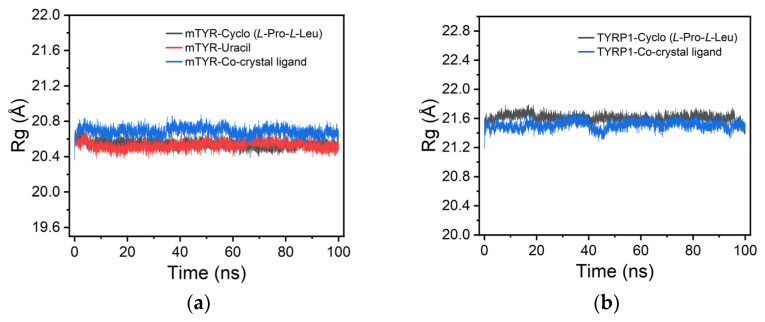
Rg analysis of protein–ligand complexes. (**a**) mTYR–ligand complexes; (**b**) TYRP1–ligand complexes.

For mTYR, radius of gyration (Rg) analysis indicated that the cyclo(*L*-Pro-*L*-Leu) and uracil complexes closely matched the co-crystallized inhibitor, with Rg values maintained between 20.4 and 20.8 Å, suggesting stable compactness throughout the simulation (Figure 10a). Similarly, for TYRP1, cyclo(*L*-Pro-*L*-Leu) also exhibited stable compactness comparable to the co-crystallized inhibitor, with Rg values ranging from 21.4 to 21.8 Å (Figure 10b).

**Figure 11:**
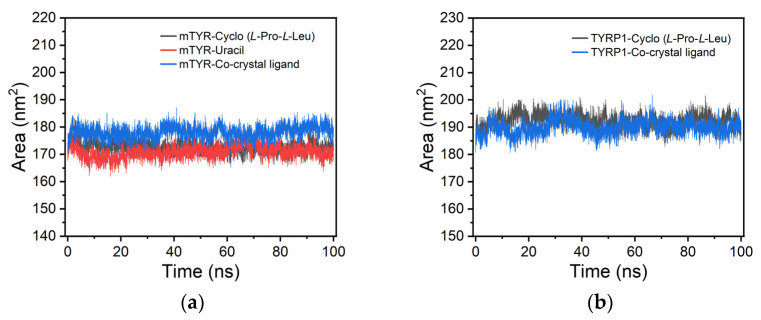
SASA analysis of protein–ligand complexes. (**a**) mTYR–ligand complexes; (**b**) TYRP1–ligand complexes.

For mTYR, solvent accessible surface area (SASA) analysis indicated that the cyclo(*L*-Pro-*L*-Leu) and uracil complexes closely matched the co-crystallized inhibitor, with SASA values maintained between 165 and 185 nm^2^, suggesting consistent solvent-exposed surface throughout the simulation (Figure 11a). Similarly, for TYRP1, cyclo(*L*-Pro-*L*-Leu) also exhibited stable solvent-accessible surface comparable to the co-crystallized inhibitor, with SASA values ranging from 180 to 200 nm^2^ (Figure 11b).

**Figure 12:**
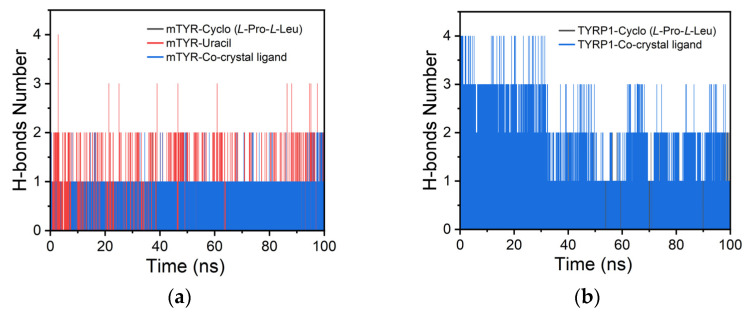
H-bonds analysis of protein–ligand complexes. (**a**) mTYR–ligand complexes; (**b**) TYRP1–ligand complexes.

For mTYR, hydrogen bond analysis indicated that cyclo(*L*-Pro-*L*-Leu) and the co-crystal inhibitor each maintained one stable hydrogen bond, whereas uracil formed two (Figure 12a). In TYRP1, cyclo(*L*-Pro-*L*-Leu) formed a single stable hydrogen bond, while the co-crystal inhibitor maintained two throughout the simulation (Figure 12b).

**Figure 13:**
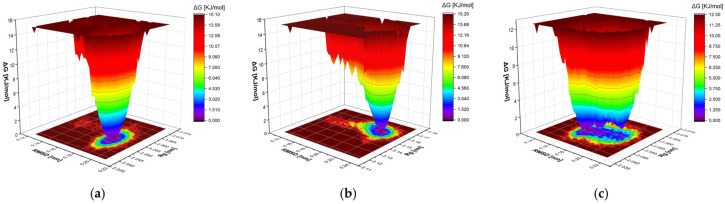
Gibbs FEL analyses of mTYR and TYRP1 complexes. (**a**) mTYR–cyclo(*L*-Pro-*L*-Leu); (**b**) mTYR–uracil; (**c**) TYRP1–cyclo(*L*-Pro-*L*-Leu).

Gibbs free energy landscape (FEL) analysis showed that the cyclo(*L*-Pro-*L*-Leu)–mTYR complex exhibited a well-defined, deep single energy basin with a free energy range of approximately 0–15.1 kJ/mol, indicating that the ligand adopts a well-constrained and stable conformation within the binding pocket (Figure 13a). In contrast, the uracil–mTYR complex had a smaller free energy range of 0–12.5 kJ/mol, but its energy landscape was flatter, more dispersed, and featured multiple local energy clusters, suggesting less confined conformations and overall lower stability (Figure 13b). For TYRP1, the cyclo(*L*-Pro-*L*-Leu)–TYRP1 complex displayed a well-defined, single energy cluster with a free energy range of approximately 0–15.2 kJ/mol, indicating a stable and well-constrained binding conformation (Figure 13c).

**Figure 14:**
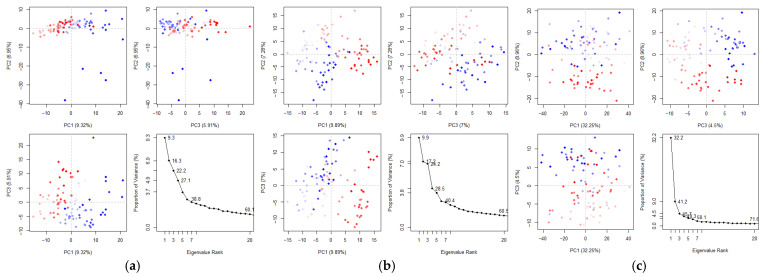

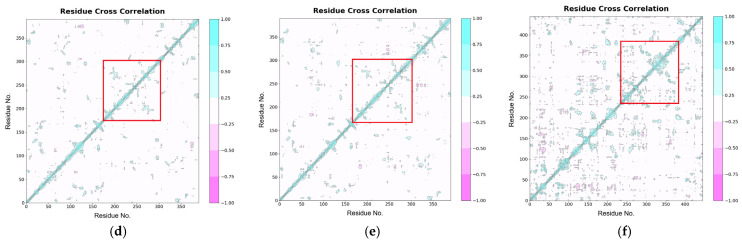
PCA and DCCM analyses of mTYR and TYRP1 complexes. PCA: (**a**) mTYR–cyclo(*L*-Pro-*L*-Leu); (**b**) mTYR–uracil; (**c**) TYRP1–cyclo(*L*-Pro-*L*-Leu). DCCM: (**d**) mTYR–cyclo(*L*-Pro-*L*-Leu); (**e**) mTYR–uracil; (**f**) TYRP1–cyclo(*L*-Pro-*L*-Leu).

Principal component analysis (PCA) analysis revealed ligand-specific modulation of collective motions in mTYR and TYRP1 (Figure 14a–c). For mTYR, both cyclo(*L*-Pro-*L*-Leu) and uracil complexes showed low variance in the first three PCs (~22–24%) and ~60% cumulative variance for the first 20 PCs, indicating broadly distributed, flexible motions with moderate ligand-induced adjustments. In TYRP1 bound to cyclo(*L*-Pro-*L*-Leu), the first three PCs accounted for ~45.7% and the first 20 PCs ~71.6% of the total variance, suggesting that a few dominant motions drive structural changes while other collective modes also contribute, reflecting intermediate flexibility and coordinated behavior.

Dynamic cross-correlation matrix (DCCM) analysis revealed ligand-dependent differences in residue–residue correlations (Figure 14d–f), where blue regions indicate positively correlated (co-directional) motions and pink regions represent negatively correlated (anti-directional) motions between residue pairs. In mTYR, both the cyclo-(*L*-Pro-*L*-Leu) and uracil complexes exhibited strong positive correlations within the active site (residues 180–350, red box), indicating that ligand binding promotes co-directional motions in this region. In contrast, TYRP1 displayed correlated motions primarily within the active site (residues 250–400, red box), with positive and negative correlations of similar magnitude, reflecting a balanced combination of cooperative and opposing residue movements.

Overall, these results indicate that, upon ligand binding, mTYR exhibits predominantly positive correlations among active-site residues, whereas TYRP1 shows balanced positive and negative correlations within the active site, reflecting ligand-specific modulation of protein conformational dynamics.

**Figure 15:**
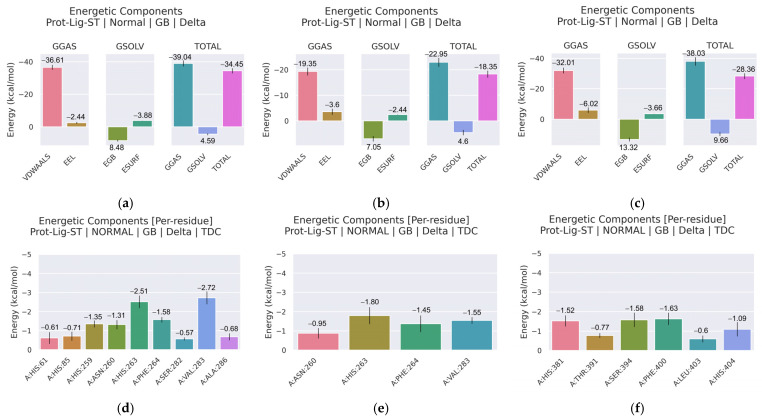
MM/GBSA binding free energies and per-residue energy decomposition of the protein–ligand complexes. (**a**,**d**) mTYR–cyclo(*L*-Pro-*L*-Leu); (**b**,**e**) mTYR–uracil; (**c**,**f**) TYRP1–cyclo(*L*-Pro-*L*-Leu).

The binding affinities of potential tyrosinase inhibitors were evaluated using MM/GBSA calculations (Figure 15a–c, Tables S4–S6). For mTYR, cyclo(*L*-Pro-*L*-Leu) exhibited a total binding free energy (ΔG total) of −34.45 kcal/mol, with van der Waals interactions making a significant contribution to the binding energy (ΔEvdw = −36.61 kcal/mol). In contrast, Uracil displayed weaker binding, with a ΔG total of −18.35 kcal/mol, consistent with the lower stability observed in MD simulations. For TYRP1, cyclo(*L*-Pro-*L*-Leu) showed a ΔG total of −28.36 kcal/mol, with van der Waals interactions contributing substantially to binding (Δ Evdw = −32.01 kcal/mol).

Previous studies have reported that, in mTYR, N81 and N260 are implicated in substrate binding, while H263 participates in π–π interactions with aromatic groups [52,53,54]. For TYRP1, prior structural analyses suggest that His381 may engage in aromatic stacking interactions, Ser394 may form hydrogen bonds, and adjacent residues contribute to ligand stabilization within the binding pocket [55]. Residue energy decomposition was performed to identify key residues contributing to the binding of potential tyrosinase inhibitors (Figure 15d–f, Tables S7–S9). For mTYR complexed with cyclo(*L*-Pro-*L*-Leu), residues His61, His85, His259, Asn260, His263, Phe264, Ser282, Val283, and Ala286 contributed favorably to the binding, with individual energies ranging from −0.57 to −2.72 kcal/mol. Val283 (−2.72 kcal/mol) and His263 (−2.51 kcal/mol) were the most significant residues in terms of their energy contribution to the complex. In comparison, Uracil binding involved fewer residues (Asn260, His263, Phe264, Val283) with weaker contributions (−0.95 to −1.80 kcal/mol), consistent with its lower overall binding affinity.

For TYRP1, cyclo(*L*-Pro-*L*-Leu) binding was stabilized by His381, Thr391, Ser394, Phe400, Leu403, and His404, with energy contributions of −1.52, −0.77, −1.58, −1.63, −0.60, and −1.09 kcal/mol, respectively. His381 (−1.52 kcal/mol), Ser394 (−1.58 kcal/mol), and Phe400 (−1.63 kcal/mol) were the most significant contributors, indicating their favorable contributions to ligand binding without implying mechanistic specificity.

Integrating prior residue-level MM/GBSA energy decomposition analyses of maculosin with the present results suggests a broadly similar energetic trend [23]. Both cyclo(*L*-Pro-*L*-Leu) and maculosin exhibit favorable contributions from His263 and Val283 in the mTYR system, as well as His381 in the TYRP1 system. Differences were also observed in the identities and energetic contributions of additional residues between the two systems. Further simulations across a broader set of bioactive cyclic dipeptides will be required to systematically evaluate how amino acid composition shapes binding energetics.

## 4. Conclusions

*Lysinibacillus* sp. JNUCC 52 was evaluated as a microbial source of tyrosinase inhibitors using an integrated strategy combining genome annotation, metabolite identification, and computational analysis. This study provides evidence that strain JNUCC 52 produces metabolites with tyrosinase inhibitory activity. Functional genomic profiling revealed a metabolism-centered architecture that supports secondary metabolite biosynthesis. Among the isolated metabolites, cyclo(*L*-Pro-*L*-Leu) was identified for the first time and exhibited the strongest inhibitory activity against tyrosinase. Mechanistically, molecular docking and MD simulations suggested that cyclo(*L*-Pro-*L*-Leu) can adopt a stable binding mode in both mTYR and TYRP1, remaining positioned within the active site during the simulations. In contrast, uracil exhibited a metal-dependent binding mode primarily mediated by monodentate carbonyl coordination, resulting in reduced conformational stability, particularly in TYRP1. MM/GBSA binding free energy calculations and residue decomposition analyses consistently supported the superior stability and affinity of cyclo(*L*-Pro-*L*-Leu). To further investigate the inhibitory mechanism, enzyme kinetic studies and melanogenesis assays in cellular models will be conducted in future work. Overall, these findings offer mechanistic insight into ligand-dependent tyrosinase inhibition, with cyclo(*L*-Pro-*L*-Leu) representing a simple natural scaffold, which may serve as a reference point for subsequent enzyme inhibition studies.

## Data Availability

The original contributions presented in this study are included in the article/Supplementary Materials. Further inquiries can be directed to the corresponding author.

## Supplementary Materials

The following supporting information can be downloaded at https://www.mdpi.com/article/10.3390/cimb48030280/s1.
