# Supplementary material for: Discovery of Tyrosinase Inhibitors from Lysinibacillus sp. JNUCC 52 via Genome Mining, Secondary Metabolites Profiling, and In Silico Analysis
Source: Curr Issues Mol Biol. 2026 Mar 5;48(3):280. doi: 10.3390/cimb48030280 (PMC13025830; doi:10.3390/cimb48030280)

Discovery of Tyrosinase Inhibitors from *Lysinibacillus* sp. JNUCC 52 via Genome Mining, Secondary Metabolites Profiling, and In Silico Analysis

Xuhui Liang [†,‡], Yang Xu [†] and Chang-Gu Hyun [*]

Department of Chemistry and Cosmetics, Jeju National University, Jeju 63243, Republic of Korea

* Correspondence: cghyun@jejunu.ac.kr; Tel.: +82-64-754-1803

† These authors contributed equally to this work.

‡ Current address: Department of Materials, Linyi Institute of Technology, Linyi 276005, China.

CONTENTS

Table S1. Analytical HPLC chromatogram of the samples under gradient elution. 1

Table S2. ADMET properties of the compounds. 1

Table S3. Drug-likeness properties of the compounds. 2

Table S4. Binding free energy analysis of the mTYR-Cyclo(*L*-Pro-*L*-Leu) complex. 2

Table S5. Binding free energy analysis of the mTYR-uracil complex. 2

Table S6. Binding free energy analysis of the TYRP1- Cyclo(*L*-Pro-*L*-Leu) complex. 2

Table S7. Residue energy decomposition analysis of the mTYR-Cyclo(*L*-Pro-*L*-Leu) complex. 3

Table S8. Residue energy decomposition analysis of the mTYR-uracil complex. 3

Table S9. Residue energy decomposition analysis of the TYRP1-Cyclo(*L*-Pro-*L*-Leu) complex. 3

Figure S1. Analytical HPLC chromatogram of the crude extract. 3

Figure S2. Analytical HPLC chromatograms of compounds **1** (**a**), **3** (**b**), **4** (**c**), and **5** (**d**). 4

Figure S3. ^{1}H NMR of compound **1** 4

Figure S4. ^{13}C NMR of compound **1** 5

Figure S5. ^{1}H NMR of compound **2** 5

Figure S6. ^{13}C NMR of compound **2** 6

Figure S7. ^{1}H NMR of compound **3** 6

Figure S8. ^{13}C NMR of compound **3** 7

Figure S9. ^{1}H NMR of compound **4** 7

Figure S10. ^{13}C NMR of compound **4** 8

Figure S11. ^{1}H NMR of compound **5** 8

Figure S12. ^{13}C NMR of compound **5** 9

Figure S13. Representative snapshots extracted from the MD simulations of the protein–ligand complexes. 10

Table S1. Analytical HPLC chromatogram of the samples under gradient elution.

The mobile phase consisted of solvent A (0.1% acetic acid in water) and solvent B (acetonitrile). Gradient elution was performed at a flow rate of 1.0 mL/min.

Time	Solution (A) %	Solution (B) %
0 to 10 min	90	10
10 to 20 min	60	40
20 to 28 min	30	70
28 to 30 min	5	95
30 to 40 min	90	10

Table S2. ADMET properties of the compounds.

CYP450: cytochrome p450; $T_{1/2}$: time required for the plasma concentration of a drug to decrease

ADMET properties		Maculosin	Cyclo(*L*-Pro-*L*-Leu)	Uracil	Arbutin
Absorption					
Caco-2 permeability (cm/s) [a]		-0.009	-2.058	-1.998	0.009
P-gp Ⅰ protein inhibitor [a]		No	No	No	No
P-gp Ⅱ protein inhibitor [a]		No	No	No	No
P-gp substrate [a]		Yes	No	No	No
Human intestinal absorption [a]		66.287%	84.241%	100%	38.027%
Distribution					
Plasma protein binding [b]		44.6%	24.1%	8.6%	54.2%
Volume distribution (L/kg) [a]		0.204	-0.104	0.089	0.026
Blood–brain barrier [c]		No	No	No	No
Metabolism					
CYP450	CYP1A2 inhibitor [c]	No	No	No	No
	CYP2C19 inhibitor [c]	No	No	No	No
	CYP2C9 inhibitor [c]	No	No	No	No
	CYP2D6 inhibitor [c]	No	No	No	No
	CYP3A4 inhibitor [c]	No	No	No	No
Elimination					
Clearance rate (log mL/min/kg) [a]		0.264	1.214	1.261	0.524
$T_{1/2}$ (h) [b]		1.398	2.653	2.314	2.361
Toxicity					
Hepatotoxicity [a]		Yes	No	No	No
Ames toxicity [a]		No	No	No	No
Skin sensitization [a]		No	No	No	No
hERG inhibition [a]		No	No	No	No
LD_{50} of acute toxicity (mol/kg) [a]		1.674	2.027	2.482	1.641

by 50%; hERG: human Ether-a-go-go-Related Gene; a: pkCSM; b: ADMETlab 3.0; c: SwissADME.

Table S3. Drug-likeness properties of the compounds.

Compound	MW[a] (g/mol)	HBA [a]	HBD [a]	RB[a]	TPSA[a] (Å^2)	Log *P*[a]	MR[b]	RO5[b]	Ghose Filter[b]	Veber rule[b]	Egan rule[b]
Maculosin	260	5	2	2	69.64	0.316	76.78	Yes	Yes	Yes	Yes
Cyclo(*L*-Pro-*L*-Leu)	210	4	1	2	49.41	1.212	64.69	Yes	Yes	Yes	Yes
Uracil	112	4	2	0	65.72	-1.102	27.68	Yes	No*	Yes	Yes
Arbutin	272	7	5	3	119.61	-0.996	62.61	Yes	No**	Yes	Yes

MW: molecular weight; HBA: number of H-Bond acceptors; HBD: number of H-Bond donors; RB: number of rotatable bonds; TPSA: Topological Polar Surface Area; MR: molar refractivity; a: ADMETlab 3.0; b: SwissADME; *: 4 violations: MW<160, WLOGP<-0.4, MR<40, atoms<20; **: one violation: WLOGP<-0.4.

Table S4. Binding free energy analysis of the mTYR-Cyclo(*L*-Pro-*L*-Leu) complex.

Frames	VDWAALS	EEL	EGB	ESURF	GGAS	GSOLV	TOTAL
Average	-36.61	-2.44	8.48	-3.88	-39.04	4.59	-34.45
SD	1.53	0.59	0.63	0.04	1.87	0.62	1.57
SEM	0.46	0.18	0.19	0.01	0.56	0.19	0.47

Table S5. Binding free energy analysis of the mTYR-uracil complex.

Frames	VDWAALS	EEL	EGB	ESURF	GGAS	GSOLV	TOTAL
Average	-19.35	-3.6	7.05	-2.44	-22.95	4.6	-18.35
SD	1.52	1.2	1.08	0.07	1.78	1.05	1.36
SEM	0.46	0.36	0.32	0.02	0.54	0.32	0.41

Table S6. Binding free energy analysis of the TYRP1- Cyclo(*L*-Pro-*L*-Leu) complex.

Frames	VDWAALS	EEL	EGB	ESURF	GGAS	GSOLV	TOTAL
Average	-32.01	-6.02	13.32	-3.66	-38.03	9.66	-28.36
SD	1.86	1.76	1.26	0.06	2.8	1.25	1.88
SEM	0.56	0.53	0.38	0.02	0.84	0.38	0.57

Table S7. Residue energy decomposition analysis of the mTYR-Cyclo(*L*-Pro-*L*-Leu) complex.

Frames	HIS:61	HIS:85	HIS:259	ASN:260	HIS:263	PHE:264	SER:282	VAL:283	ALA:286
Average	-0.61	-0.71	-1.35	-1.31	-2.51	-1.58	-0.57	-2.72	-0.68
SD	0.31	0.24	0.18	0.24	0.33	0.14	0.08	0.34	0.18
SEM	0.09	0.07	0.05	0.07	0.1	0.04	0.02	0.1	0.05

Table S8. Residue energy decomposition analysis of the mTYR-uracil complex.

Frames	ASN:260	HIS:263	PHE:264	VAL:283
Average	-0.95	-1.80	-1.45	-1.55
SD	0.26	0.44	0.43	0.17
SEM	0.08	0.13	0.13	0.05

Table S9. Residue energy decomposition analysis of the TYRP1-Cyclo(*L*-Pro-*L*-Leu) complex.

Frames	HIS:381	THR:391	SER:394	PHE:400	LEU:403	HIS:404
Average	-1.52	-0.77	-1.58	-1.63	-0.6	-1.09
SD	0.28	0.12	0.37	0.31	0.17	0.37
SEM	0.09	0.04	0.11	0.09	0.05	0.11

Figure S1. Analytical HPLC chromatogram of the crude extract.

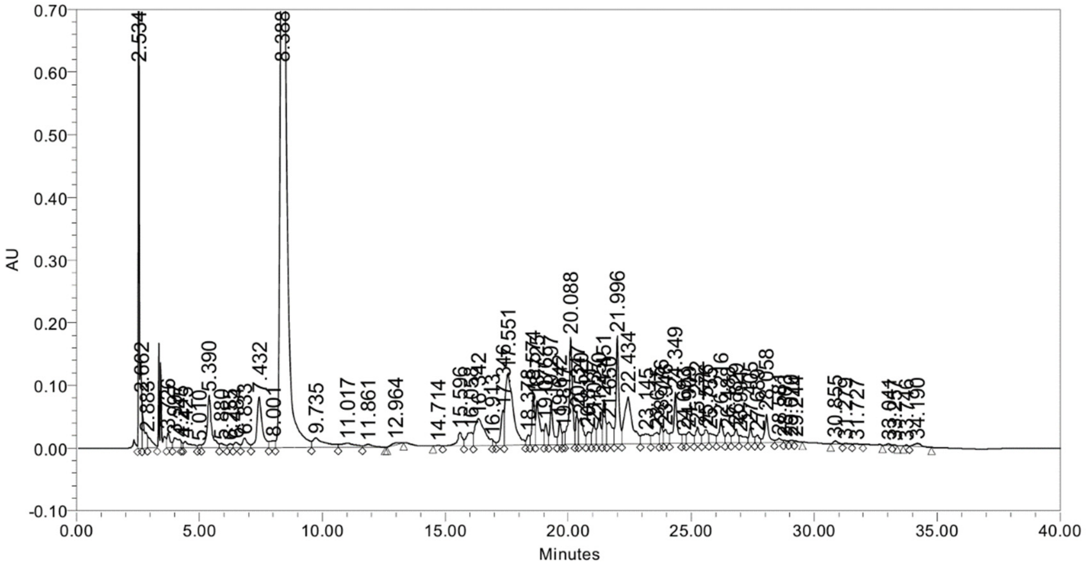

Figure S2. Analytical HPLC chromatograms of compounds **1** (**a**), **3** (**b**), **4** (**c**), and **5** (**d**).

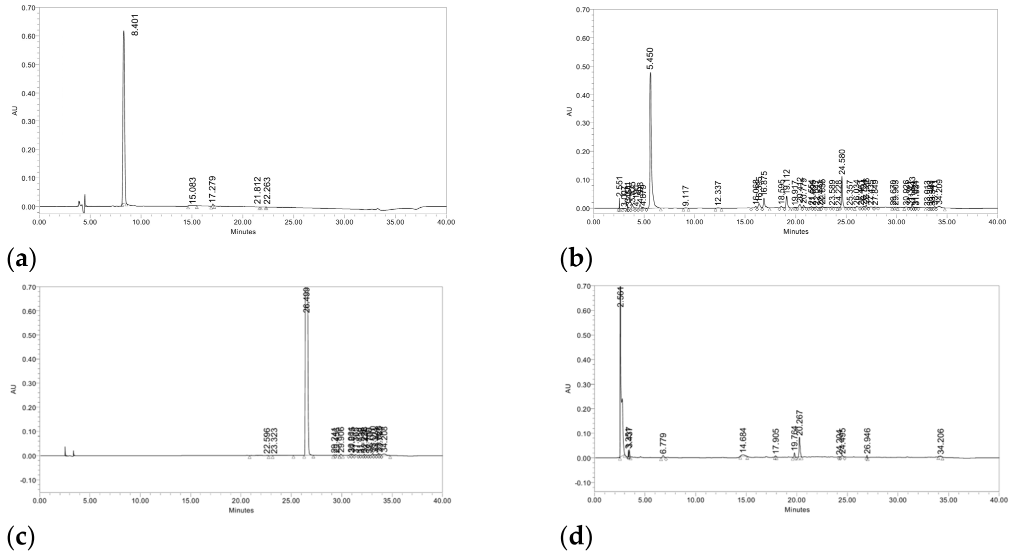

Figure S3. ^{1}H NMR of compound **1**

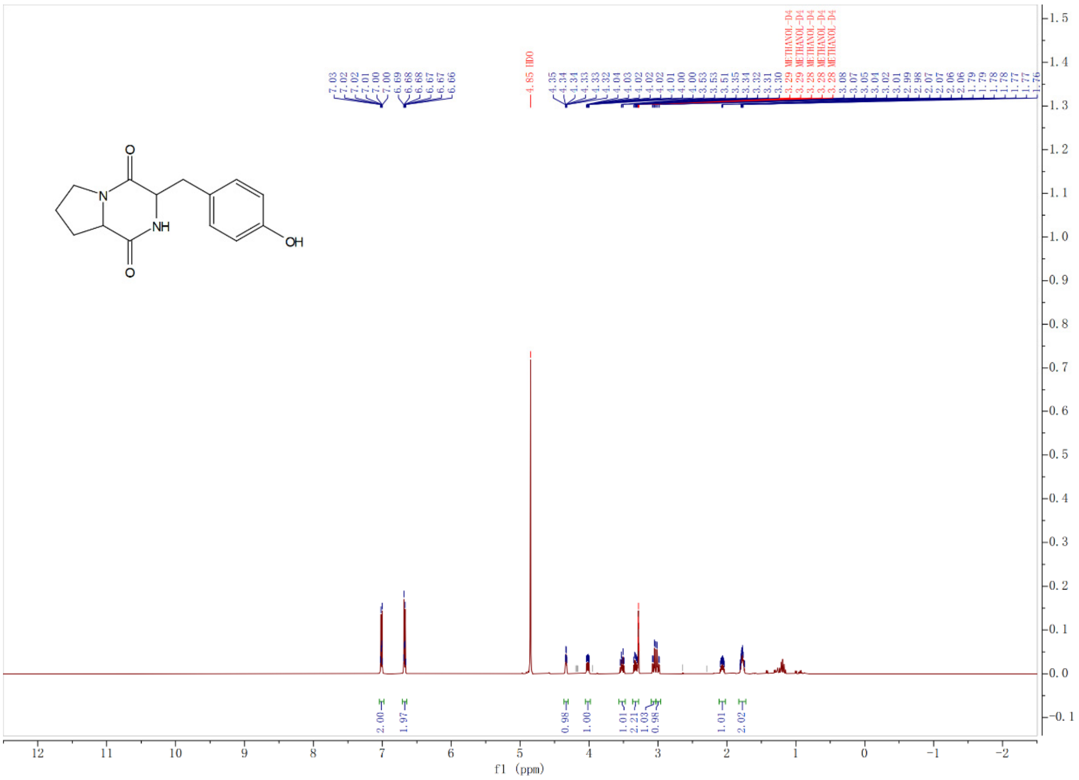

Figure S4. ^{13}C NMR of compound **1**

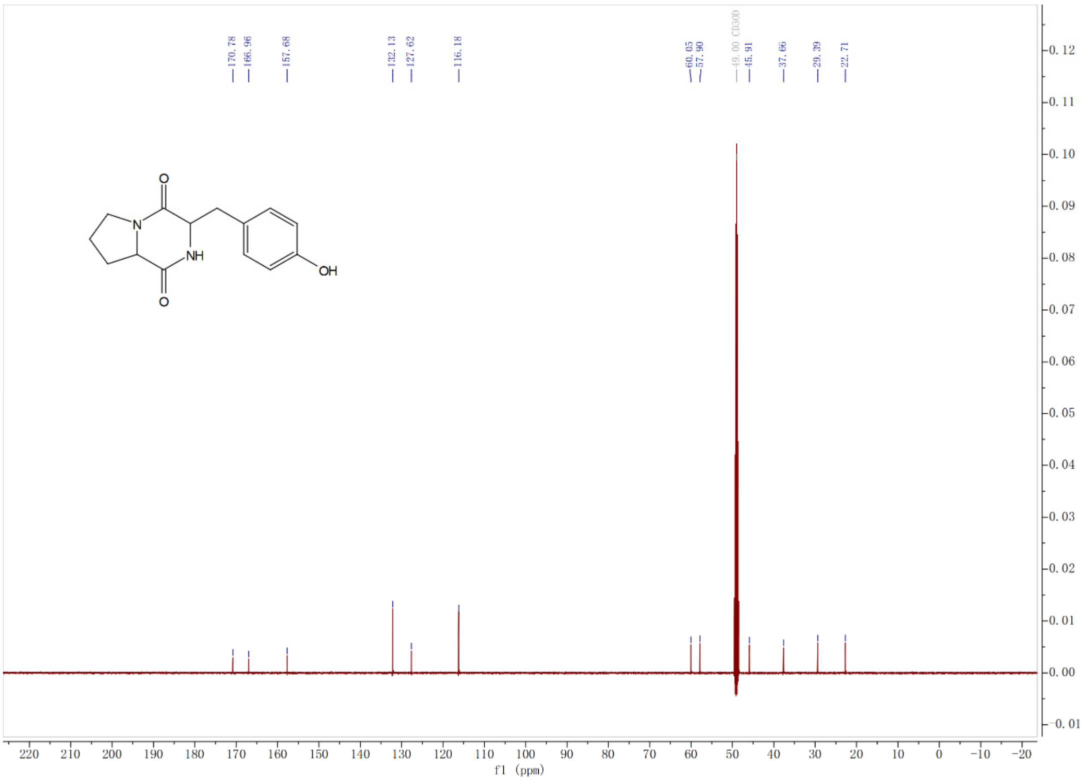

Figure S5. ^{1}H NMR of compound **2**

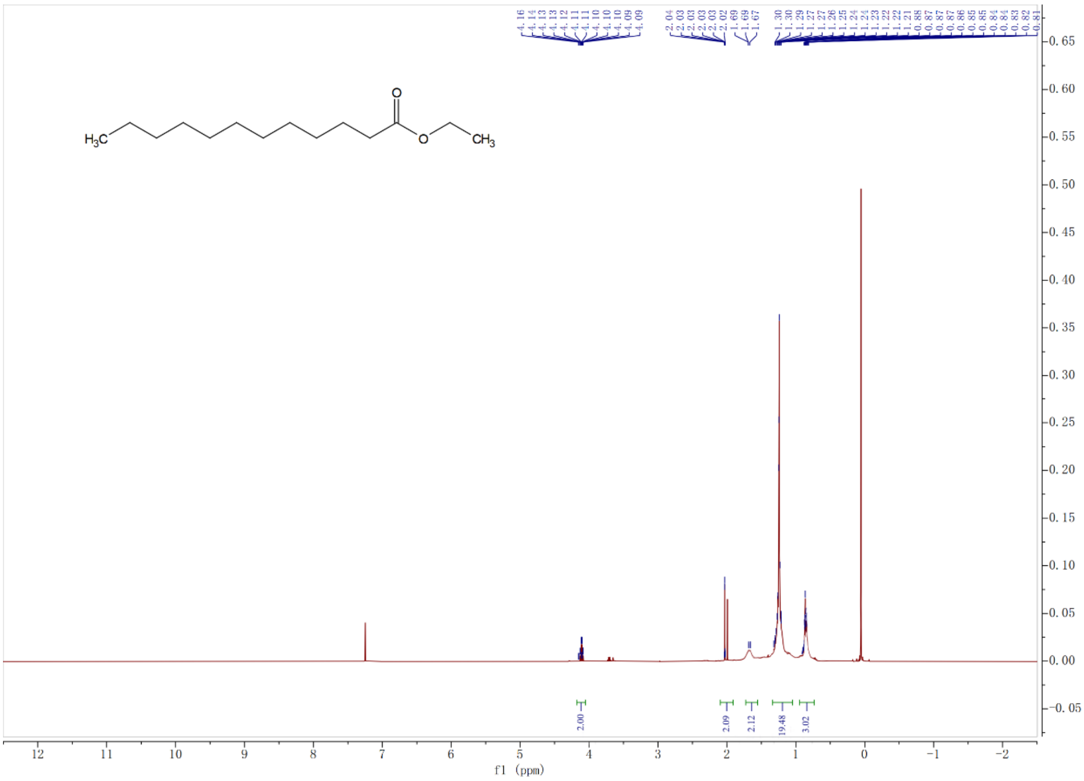

Figure S6. ^{13}C NMR of compound **2**

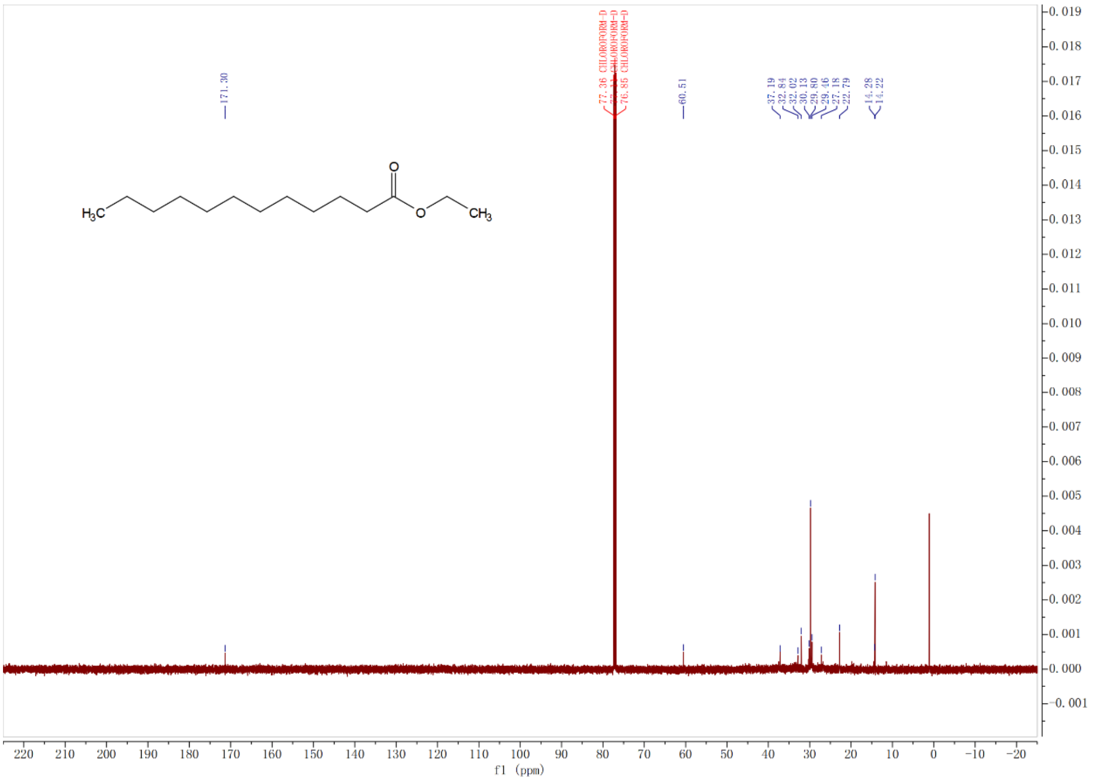

Figure S7. ^{1}H NMR of compound **3**

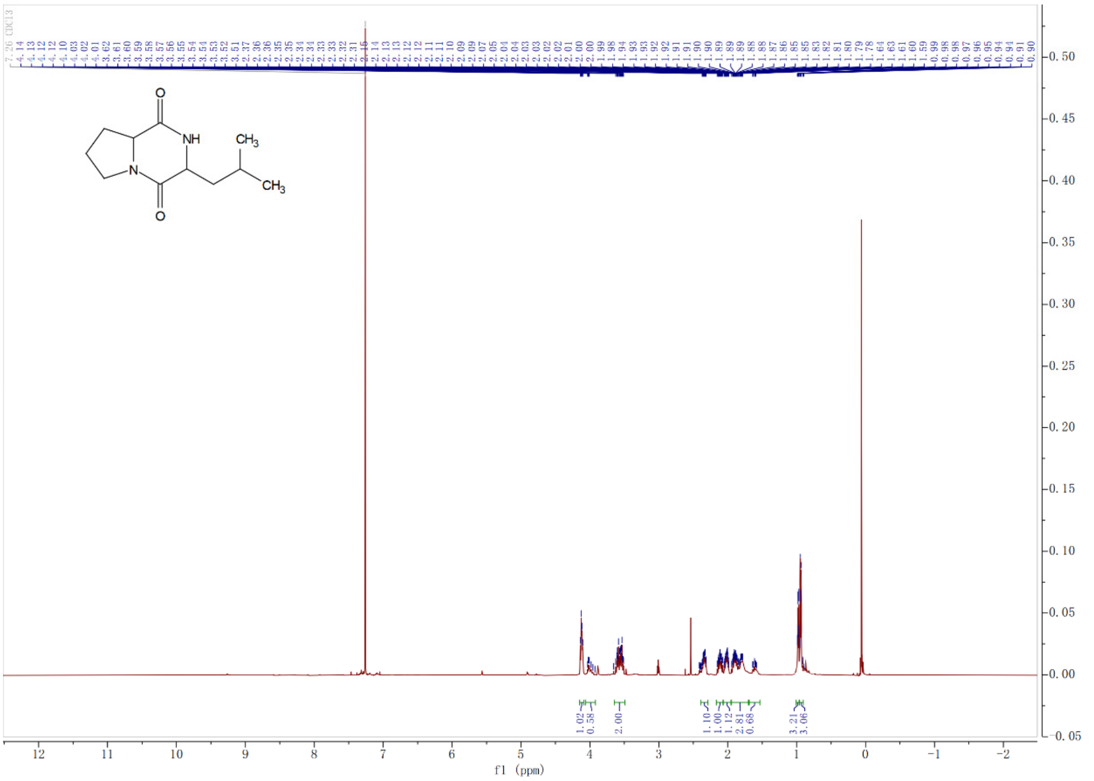

Figure S8. ^{13}C NMR of compound **3**

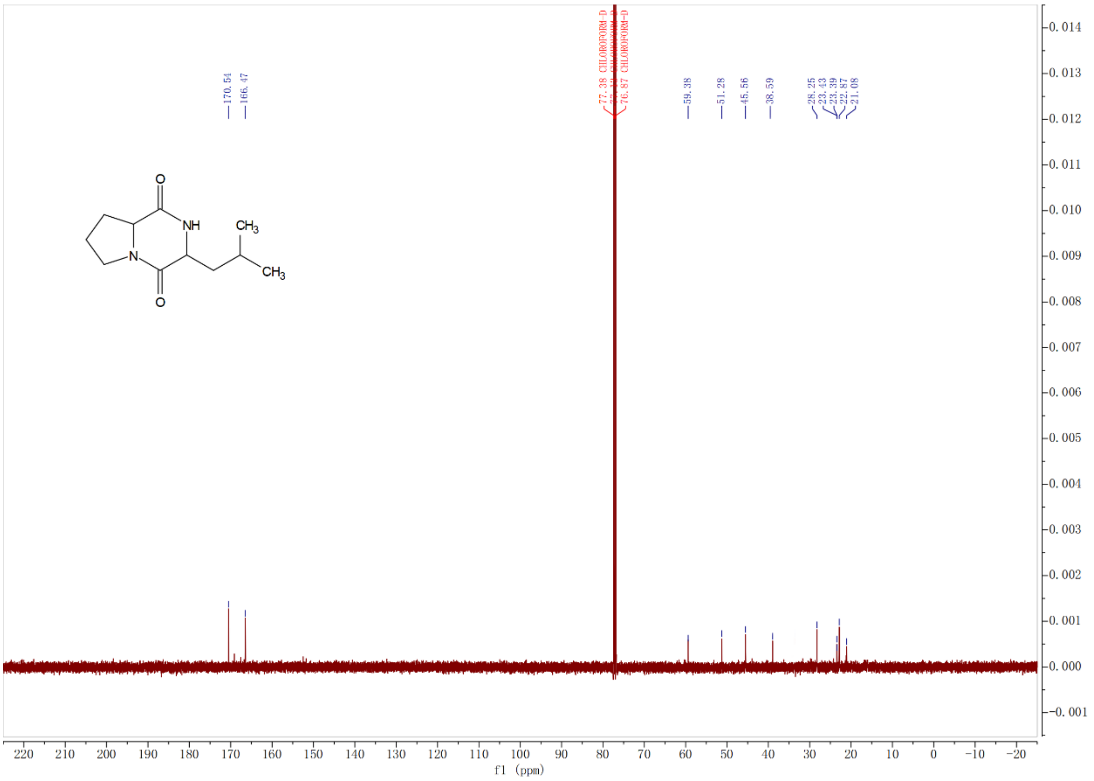

Figure S9. ^{1}H NMR of compound **4**

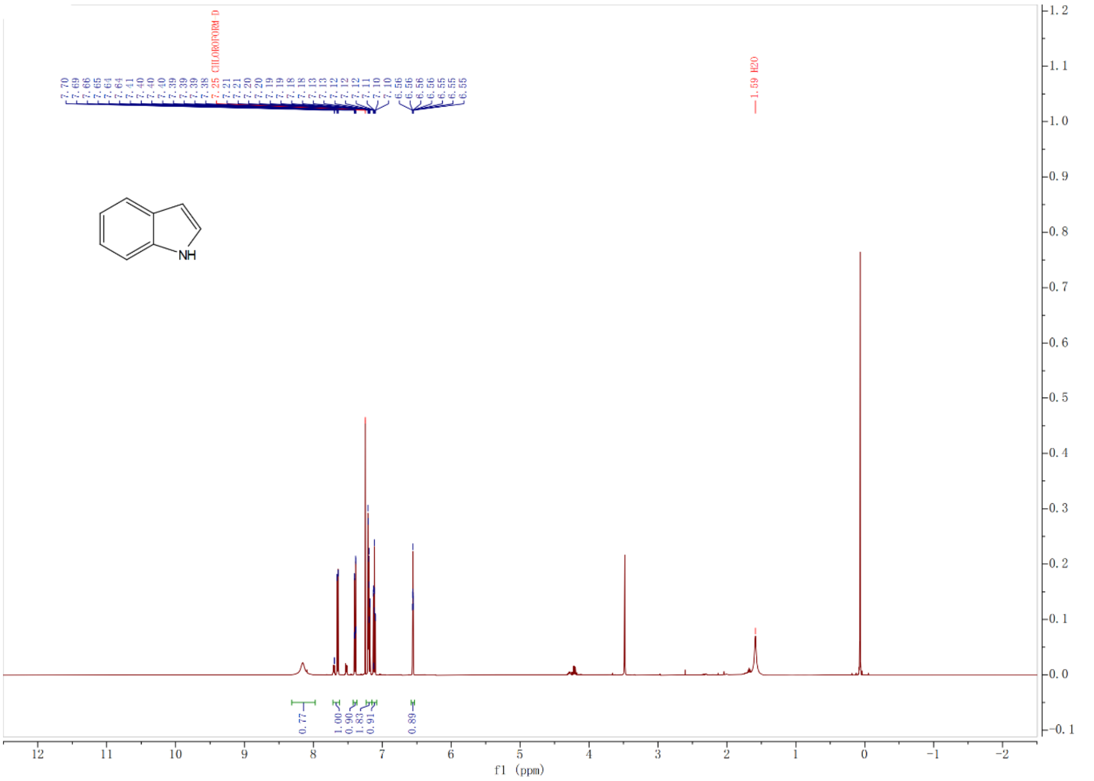

Figure S10. ^{13}C NMR of compound **4**

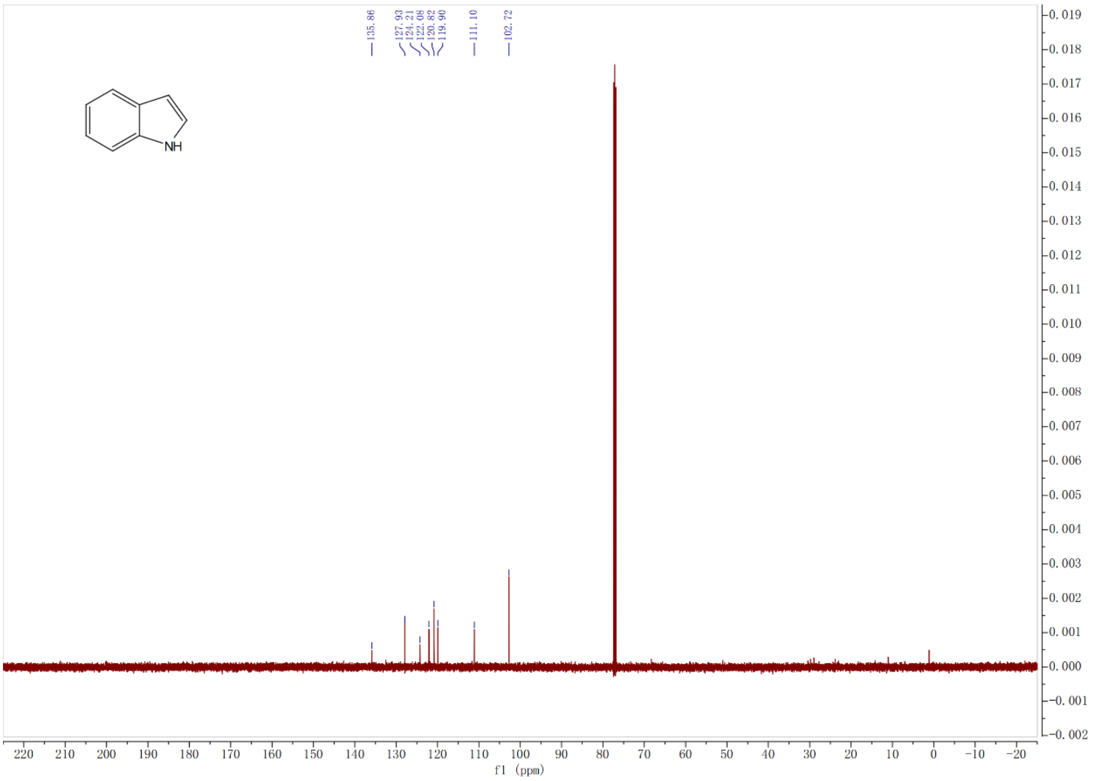

Figure S11. ^{1}H NMR of compound **5**

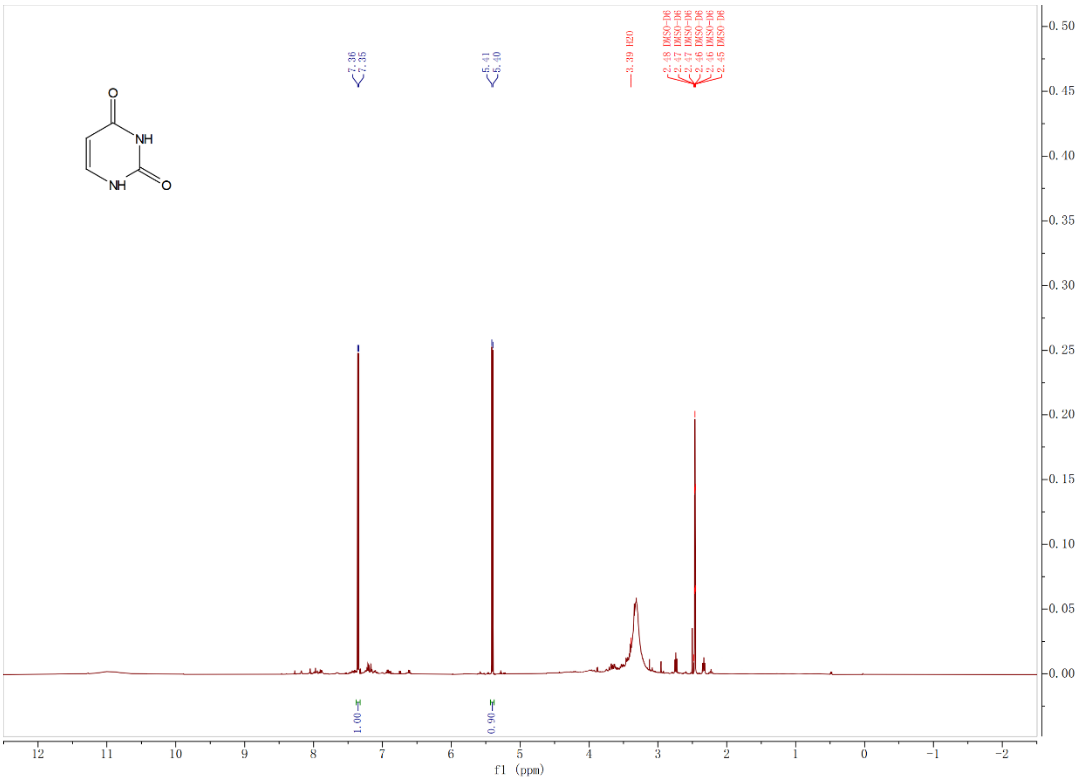

Figure S12. ^{13}C NMR of compound **5**

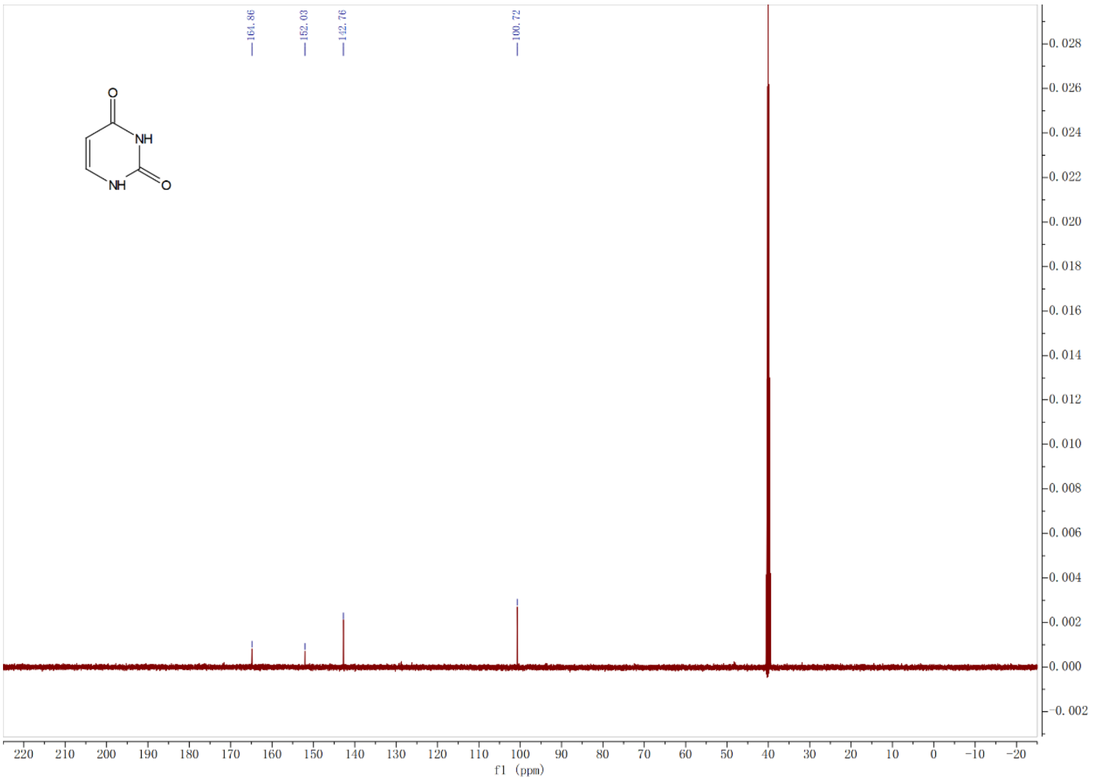

Figure S13. Representative snapshots extracted from the MD simulations of the protein–ligand complexes.

Snapshots at 0, 25, 50, 75, and 100 ns are shown in green, blue, purple, yellow, and pink, respectively. The active-site region is highlighted by red dashed outlines.

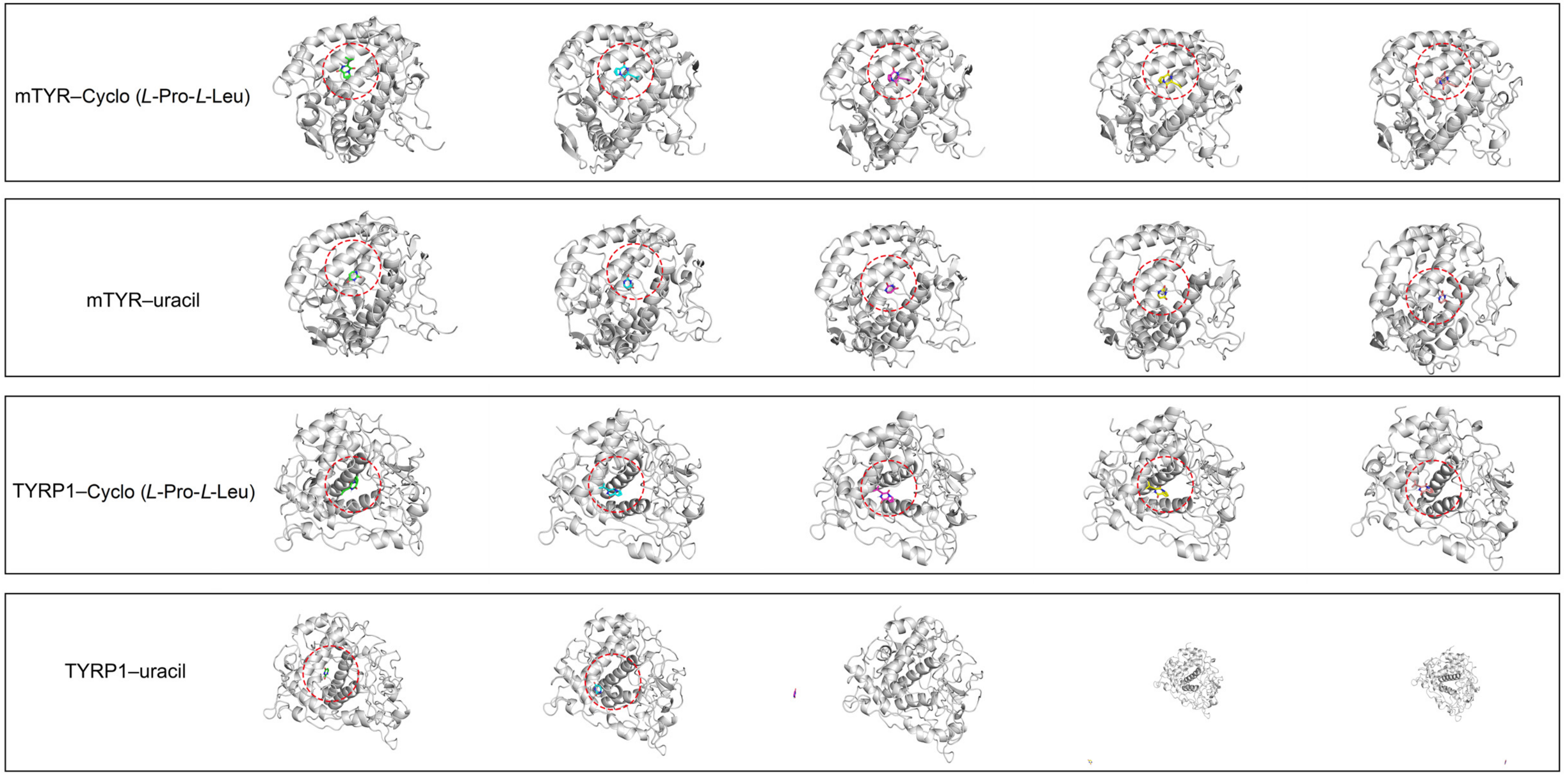

Supplement: Supplementary file 1 [file cimb-48-00280-s001.zip › cimb-4156538-supplementary.pdf]
